# Characterization of methionine oxidation and methionine sulfoxide reduction using methionine-rich cysteine-free proteins

**DOI:** 10.1186/1471-2091-13-21

**Published:** 2012-10-23

**Authors:** Xinwen Liang, Alaattin Kaya, Yan Zhang, Dung Tien Le, Deame Hua, Vadim N Gladyshev

**Affiliations:** 1 Department of Biochemistry and Redox Biology Center, University of Nebraska, Lincoln, NE, 68588, USA; 2 Division of Genetics, Brigham and Women’s Hospital and Harvard Medical School, 77 Avenue Louis Pasteur, Boston, MA, 02115, USA; 3 Key Laboratory of Systems Biology, Shanghai Institutes for Biological Sciences, Chinese Academy of Sciences, Shanghai, 200031, China

**Keywords:** Methionine, Methionine sulfoxide, Cysteine, Methionine sulfoxide reductase, Protein oxidation and reduction, Protein repair, Antibodies

## Abstract

**Background:**

Methionine (Met) residues in proteins can be readily oxidized by reactive oxygen species to Met sulfoxide (MetO). MetO is a promising physiological marker of oxidative stress and its inefficient repair by MetO reductases (Msrs) has been linked to neurodegeneration and aging. Conventional methods of assaying MetO formation and reduction rely on chromatographic or mass spectrometry procedures, but the use of Met-rich proteins (MRPs) may offer a more streamlined alternative.

**Results:**

We carried out a computational search of completely sequenced genomes for MRPs deficient in cysteine (Cys) residues and identified several proteins containing 20% or more Met residues. We used these MRPs to examine Met oxidation and MetO reduction by in-gel shift assays and immunoblot assays with antibodies generated against various oxidized MRPs. The oxidation of Cys-free MRPs by hydrogen peroxide could be conveniently monitored by SDS-PAGE and was specific for Met, as evidenced by quantitative reduction of these proteins with Msrs in DTT- and thioredoxin-dependent assays. We found that hypochlorite was especially efficient in oxidizing MRPs. Finally, we further developed a procedure wherein antibodies made against oxidized MRPs were isolated on affinity resins containing same or other oxidized or reduced MRPs. This procedure yielded reagents specific for MetO in these proteins, but proved to be ineffective in developing antibodies with broad MetO specificity.

**Conclusion:**

Our data show that MRPs provide a convenient tool for characterization of Met oxidation, MetO reduction and Msr activities, and could be used for various aspects of redox biology involving reversible Met oxidation.

## Background

Methionine (Met) is a sulfur-containing amino acid that is particularly susceptible to oxidation by reactive oxygen species (ROS). Methionine oxidation results in the formation of a mixture of two diastereomeric forms of methionine sulfoxide (MetO), which can be reduced back to Met by methionine sulfoxide reductases (Msrs) A (MsrA) and B (MsrB). These enzymes play an important role in protection of proteins against oxidative stress and have been implicated in regulation of the aging process. Insufficient repair of oxidized Met by these enzymes may alter protein structure and cause protein aggregation and/or loss of function, accelerating age-associated diseases [1-10]. Higher levels of MetO and carbonyl groups in proteins were found in the brains of Alzheimer’s disease patients based on postmortem pathology analyses [11]. The reversible oxidation of Met residues may also modulate protein-protein interactions and participate in intracellular signaling [12,13]. Thus, identification of proteins most susceptible to Met oxidation may help better understand age-associated diseases and signaling pathways.

Conventional assays for detection of MetO and monitoring its reduction rely on HPLC and mass-spectrometry procedures [14,15]. In addition, there have been several reports on the use of antibodies specific for certain MetO-containing peptides to detect oxidation of these peptides [13,16]. There is also a report on the use of antibodies specific for oxidized plant protein DZS18, enriched for Met residues, to detect MetO in cellular proteins [17]. While providing new tools and approaches, these studies, as well as our own previous analyses, have not yet resulted in specific identification of MetO in proteins under physiological conditions.

In this regard, an important approach in developing tools for MetO research is the use of Met-rich proteins (MRPs). Previously, we identified and characterized three such proteins and developed methods to monitor their Met oxidation and MetO reduction by SDS-PAGE and immunoblot assays [18]. In the current work, we describe four new MRPs, which in addition to Met enrichment have an added benefit of being deficient in cysteine (Cys), another sulfur-containing amino acid highly susceptible to oxidation. We report characterization of these proteins and antibodies generated against MetO forms of these proteins. We further used these MRPs to examine various features of Met oxidation and repair in proteins.

## Methods

*Materials*. Genomic DNA of *Idiomarina loihiensis* L2TR and *Saccharophagus degradans* 2–40 was kindly provided by Dr. Maqsudul Alam and Dr. Steven Hutcheson, respectively. *Pseudomonas putida* W619 cells [19] were kindly provided by Dr. Daniel van der Lelie. Expression constructs for MRP1 and MRP2, mouse MsrA, mouse MsrB2, and MsrA-MsrB fusion protein (MsrBA) were previously described [18,20,21]. Msr null strains were also described previously [22]. Reagents used in this work were of the highest grade possible.

### Computational identification of MRPs

In an initial step of the search procedure, we adopted a strategy similar to that previously used for identification of MRPs [18]. Specifically, using in-house scripts, each protein in the NCBI non-redundant protein dataset (downloaded from ftp://ftp.ncbi.nih.gov) was scanned for Met content and protein length. Sequences with Met content above 20%, length more than 50 amino acids and pronounced secondary structures, were selected. Subsequently, Cys content was analyzed for each MRP. Proteins with more than 20% Met and low number of Cys (or lacking Cys) were further considered. The MRP candidates deemed more likely to be soluble were then selected by manual analysis for further experimental studies.

### Cloning, expression, purification and mass spectrometry analyses of MRPs

Sequences coding for MRP4 (from codon 23 to stop codon), MRP5 (from codon 21 to stop codon), and MRP6 (from codon 21 to stop codon) were PCR-amplified from the corresponding genomic DNA. MRP3 coding region starting from amino acid 22 was amplified from mRNA isolated from *Zea mays*. MRP7 coding region was amplified from mouse liver mRNA. PCR products of MRP3-MRP6 were sequenced, digested with either NdeI and NotI or Nhe1 and Not1 (for MRP4, MRP6) and cloned into pET21b vector to generate the constructs coding for proteins with His-tags at the C-terminus. The PCR product of MRP7 was cloned into Nde1/Not1 sites of pET28a+ to generate the construct coding a protein with a His-tag at both N- and C-terminus. Soluble (MRP1-MRP2, MRP4-MRP7) and insoluble (MRP3) recombinant MRPs were purified on Talon columns following expression in *E. coli* using a previous procedure [18]. Mass-spectrometry analyses of MRPs were also carried out as described previously [18].

### Oxidation of MRPs by H_2_O_2_

Purified MRPs were subjected to controlled oxidation by various concentrations of H_2_O_2_ or other indicated oxidants, wherein oxidation was monitored by the mobility of proteins on SDS-PAGE gels. To verify that the shift in mobility was due to Met oxidation, the oxidized proteins were subjected to reduction with Msrs in the presence of DTT or thioredoxin (Trx) as described below.

### Preparation of oxidized MRPs to generate antibodies specific for oxidized MRPs

Purified MRP3-MRP7 were oxidized with 10–50 mM H_2_O_2_ at room temperature for 12 h, the oxidized proteins subjected to SDS-PAGE analysis to determine the extent of oxidation, and ESI-MS analyses were carried out to measure the number of oxidized Met residues. The conditions that resulted in oxidation of all Met in proteins (these were slightly different for various MRPs) were used to prepare oxidized MRPs at large scale for immunization. Rabbit polyclonal antibodies were produced by Covance, Inc.

### Enrichment of antibodies specific for oxidized MRPs

Affinity columns containing reduced or oxidized MRP were used to enrich polyclonal antibodies specific for oxidized MRPs. To prepare an MRP affinity column, 2–6 mg of a reduced or oxidized MRP (the same preparation as that used for immunization) were coupled to NHS-activated Sepharose (GE Life Science, Piscataway, NJ) according to manufacturer’s instructions. The antibody purification procedure included the following two alternatives: (i) One-step enrichment. The antisera were first purified on a protein G Sepharose column (GE Life Science, Piscataway, NJ), and then the resulting IgG fraction (10–20 mg) was dialyzed against PBS and further applied to a column containing a reduced MRP at room temperature for 2 h, with shaking. The unbound fraction was collected and the Sepharose column was washed with 5 volumes of PBS, followed by elution of the bound antibodies. The unbound and wash fractions were re-purified on the column with reduced MRP twice using the same procedure. The final unbound and wash fractions were used for immunoblot analyses to examine specificity of the resulting antibodies. (ii) Two-step enrichment. The final unbound and wash fractions obtained by the procedure described above were further purified on the affinity columns containing oxidized MRPs and the bound antibodies were eluted and used for further immunoblot analyses.

### Msr activity assays using MRPs

The DDT-dependent assay was carried out as following: 2–4 μg of an oxidized MRP were incubated with 5 mM DTT and 0.5-4 μM Msrs at 37°C for 2 h, and the reaction mixture was mixed with SDS-PAGE sample buffer and subjected to SDS-PAGE analysis. The Trx-dependent assay was as follows: 2–4 μg of an oxidized MRP were incubated with 6.8 μM Trx, 0.2 μM NADPH, and 0.4 μM Trx reductase at 37°C for 2 h, and the reaction was subjected to SDS-PAGE analysis.

## Results

### Computational identification of MRPs

The three first-generation MRPs characterized in our previous work [18] contained Cys residues, which either interfered with some redox assays that targeted Met oxidation and MetO reduction, or required an additional blocking step prior to MetO analyses. To address this issue, we conducted a computational search for MRPs that had few or no Cys residues. An analysis of the NCBI non-redundant protein database, which also included ORFs encoded in completely sequenced genomes, revealed approximately 170 sequences with more than 20% Met content (10 fold higher Met content than expected by chance based on Met content of proteins in the NCBI database), which were present in organisms ranging from bacteria to mammals. These proteins were further analyzed for Cys content and secondary structure composition. We selected proteins expected to be soluble based on their predicted secondary structure or modeled 3D structures, and which in addition showed a maximal Met content and few or no Cys residues. The final set of MRPs selected based on this procedure had 21-33% Met and 0-2% Cys (Table 1).

**Table 1:** MRPs characterized in this study

**Protein**	**Organism**	**Accession number**	# of Met residues *	**% of Met**	# of Cys residues*	**% of Cys**
Hypothetical protein (FeNos, MRP1)	*Nostoc* sp. PCC 73102	ZP_00345849	41 (38)	33	15 (15)	12
Hypothetical protein (LegP, MRP2)	*Legionella pneumophila*	YP_095088	37 (35)	25	2 (1)	1
Delta zein structural18 (DZS18, MRP3)	*Zea mays*	NP_001105054	50 (49)	24	4 (3)	2
Hypothetical protein (MRP4)	*Pseudomonas putida* W619	YP_001746901	32 (31)	21	1 (0)	1
Hypothetical protein (MRP5)	*Idiomarina loihiensis* L2TR	YP_155605	32 (31)	22	0 (0)	0
Hypothetical protein (MRP6)	*Saccharophagus degradans* 2-40	YP_527410	36 (35)	25	0 (0)	0
Calmodulin (MRP7)	*Mus musculus*	NP_031615	10 (12)	7	0 (0)	0

* Numbers correspond to the number of indicated amino acids encoded in the ORF, and the numbers in brackets correspond to the amino acid residues present in recombinant MRPs (difference is due to removal of signal peptides).

### Preparation of recombinant MRPs

**Figure 1:**
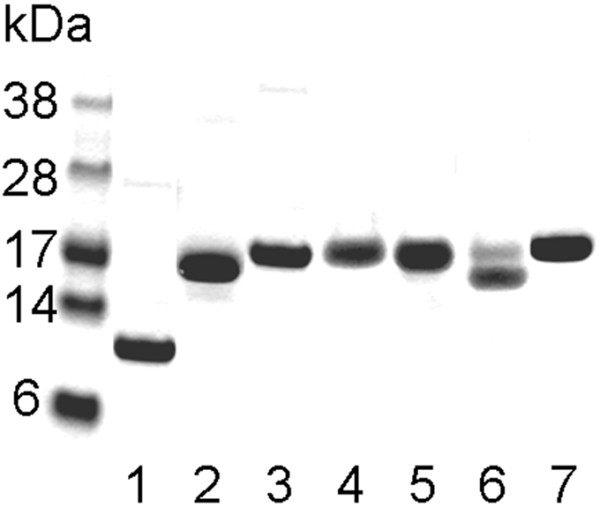
**SDS-PAGE analysis of recombinant MRPs.** His-tagged recombinant MRPs (MRP1 to MRP7) were identified through a computational procedure, cloned, expressed in *E. coli* BL21 cells, purified on Talon resin, and the purified proteins subjected to SDS-PAGE analysis and visualized by Coomassie Blue staining. Lanes 1 to 7 correspond to MRP1 to MRP7, respectively.

For experimental analyses of MRPs, we selected two proteins from a previous study, including FeNos (herein designated MRP1) from *Nostoc sp*, and LegP (herein designated MRP2) from *Legionella.* Recombinant MRP1 contained 15 Cys residues and MRP2 a single Cys residue. Both proteins were prepared as His-tag forms and found to be soluble. The new MRPs included DZS18 (here designated MRP3), a previously characterized storage protein from *Zea mays*[17], and three hypothetical proteins from *Pseudomonas putida* W619 (designated MRP4), *Idiomarina loihiensis* L2TR (MRP5), and *Saccharophagus degradans* 2–40 (MRP6). In addition, we analyzed calmodulin, a calcium sensor protein that contains regulatory Met residues [23]. Mouse calmodulin (designated MRP7) had 7% Met, so even though its Met content was below 20%, it was much higher than the average Met content of proteins (~2%). The four expressed proteins, MRP3-MRP6, were prepared in the forms that lacked predicted N-terminal signal peptides (20–22 amino acids). With regard to Cys content, MRP3 had 3 Cys residues, whereas all other new MRPs lacked Cys. These proteins could be expressed and isolated using standard procedures with a high degree of purity (Figure 1).

### MRP-based analyses of Met oxidation and MetO reduction by SDS-PAGE

**Figure 2:**
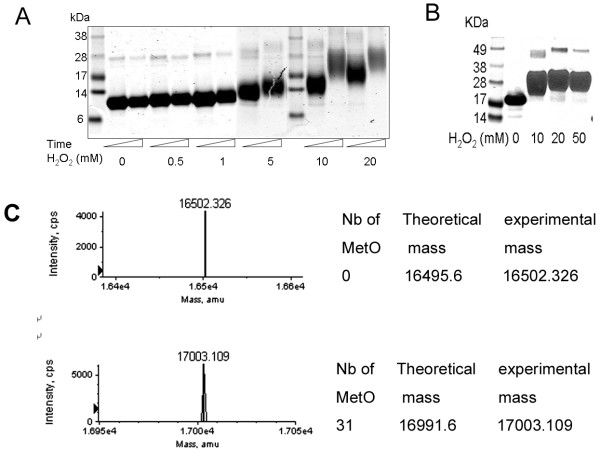
**Analyses of Met oxidation and MetO reduction in MRPs by SDS-PAGE.** (**A**) 60 μM MRP1 was treated with indicated concentrations of H_2_O_2_ for 7 or 12 h, and protein oxidation was monitored by the mobility shift on SDS-PAGE gels, as visualized by Coomassie Blue staining. Open triangles represent increasing treatment time (i.e., 7, 12 h). (**B**) 60 μM MRP5 was oxidized with indicated concentrations of H_2_O_2_ at room temperature for 12 h, and MetO formation was monitored by the mobility shift on SDS-PAGE gels, as visualized by Coomassie Blue staining. (**C**) Mass-spectrometry analysis of reduced MRP5 and the protein oxidized by 10 mM H_2_O_2_ at room temperature for 12 h. Theoretical and observed masses are shown.

We previously demonstrated that oxidation of Met residues in the first-generation, Cys-containing MRPs could be monitored by protein mobility shift using SDS-PAGE gels, and that MetO-containing MRPs migrated slower than the corresponding reduced proteins [18]. To further examine the utility of SDS-PAGE analysis for monitoring MRP oxidation, we oxidized 60 μM MRP1 with different concentrations of H_2_O_2_ and for various periods of time. The as-isolated and oxidized forms of MRP1 were resolved on 10% SDS-PAGE gels and visualized by Coomassie blue staining (Figure 2A). Migration of MRP1 only slightly changed when the protein was incubated with 1 mM or less H_2_O_2_ for 7–12 h, whereas it migrated significantly slower at higher H_2_O_2_ concentrations. Moreover, mobility of MRP1 proportionally decreased with the increase in H_2_O_2_ levels and time of treatment; the maximal oxidation was achieved by treating the protein with 10 mM H_2_O_2_ for 12 h.

We further examined Met oxidation by H_2_O_2_ in other MRPs by in-gel assays as well as by mass spectrometry. Met residues could be fully oxidized in MRP2, MRP4, MRP5, and MRP6 when 60 μM proteins were treated with 10 mM H_2_O_2_ at room temperature for 12 h; however, 20 mM and 50 mM H_2_O_2_ was required to fully oxidize MRP3 and MRP7, respectively. These data suggest that structural and sequence context in which Met is present may influence efficiency of its oxidation. Figure 2B shows oxidation of MRP5 as monitored by in-gel assays, and Figure 2C, for comparison, oxidation of the same protein as monitored by mass spectrometry. Following 10 mM H_2_O_2_ treatment, the observed mass of MRP5 increased to that corresponding to the mass of the fully oxidized MRP containing 31 MetO. Thus, Met oxidation in MRPs resulted in slower migration of all MRPs on SDS-PAGE gels, indicating that this is a general feature of Met oxidation.

### Met residues are sensitive to two-electron oxidants

**Figure 3:**
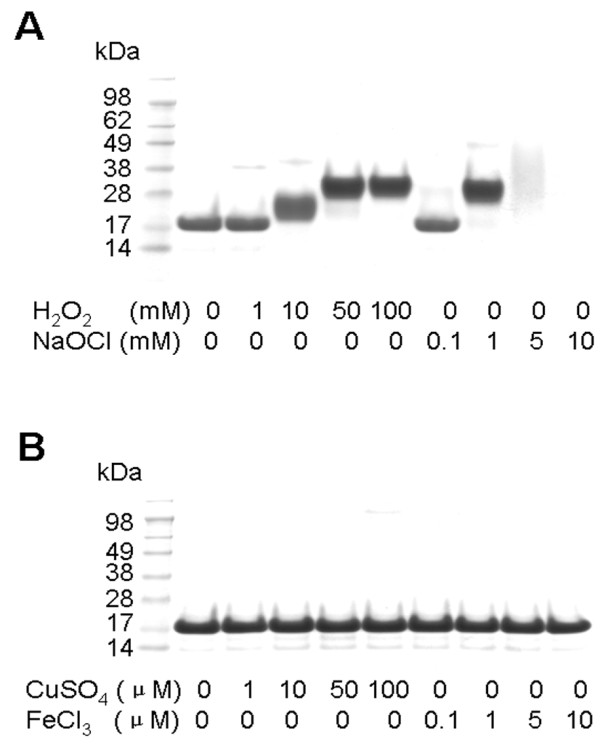
**Oxidation of Met in MRPs by various oxidants.** 20 μM MRP5 was oxidized with the indicated concentrations of (**A**) hydrogen peroxide (H_2_O_2_) or sodium hypochlorite (NaOCl) at 37°C for 1 h; (**B**) CuSO_4_ or FeCl_3_ at 37°C for 2 h, and then subjected to SDS-PAGE analysis.

To examine sensitivity of Met residues to different oxidants, we treated 20 μM MRP5 with H_2_O_2_ or NaOCl, which are two-electron oxidants, and with CuSO_4_ or FeCl_3_, which are one-electron oxidants. Treatment of MRP5 for 1 h at 37°C oxidized approximately 50% Met residues when 10 mM H_2_O_2_ was used, and full oxidation was achieved upon treatment with 50 mM H_2_O_2_. Further increase in H_2_O_2_ levels to 100 mM did not change MRP5 mobility on SDS-PAG gels, but also did not lead to significant protein degradation (Figure 3A). 1 mM NaOCl could oxidize this MRP to the same extent as that caused by the treatment with 10 mM H_2_O_2_; however the amount of oxidized MRP5 decreased when the protein was treated with 5 mM NaOCl, and the protein was essentially undetectable after treatment with 10 mM NaOCl (Figure 3A). Thus, compared with H_2_O_2_, NaOCl is a stronger oxidant of Met in proteins, and at higher concentrations it can break peptide bonds. The mobility of MRP5 on SDS-PAGE gels did not change when the protein was treated with CuSO_4_ or FeCl_3_, suggesting that they are inefficient Met oxidants (Figure 3B).

### Analyses of Msr activities by in-gel assays using MRPs as substrates

**Figure 4:**
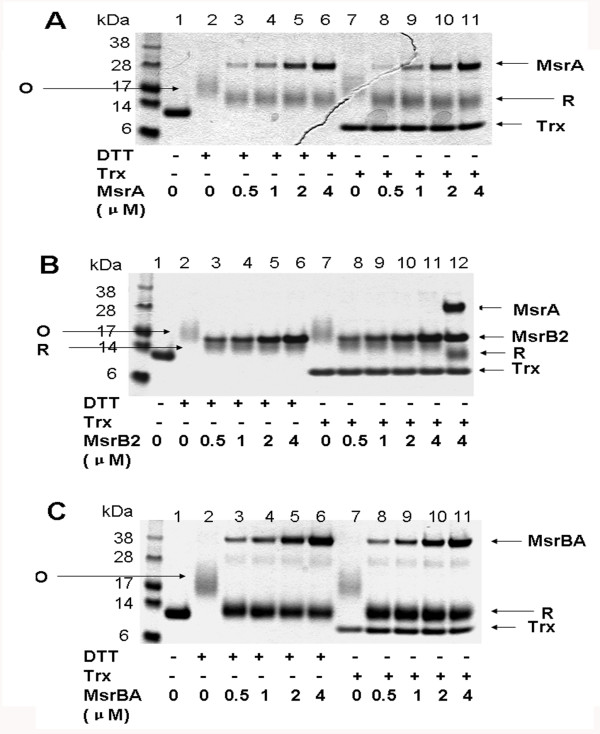
**Msr activity assays by following MRP electrophoretic mobility shift on SDS-PAGE gels.** 4 μg of as-isolated (lane 1) or oxidized (lanes 2 to 12) MRP1 were incubated with Msrs and DTT or Trx as described in Materials and Methods, electrophoresed on 10% SDS-PAGE gels and stained with Coomassie Blue. Migration of reduced (R) and oxidized (O) MRP1 is shown by arrows. (**A**) MsrA activity assay. (**B**) MsrB activity assay (lanes 2 to 11) and MsrA plus MsrB activity assay (lane 12). (**C**) MsrBA activity assay. When used separately, MsrA and MsrB partially reduced oxidized Met residues, whereas treatment with MsrBA completely restored mobility of the reduced protein.

We further subjected oxidized MRPs to treatment with Msr enzymes (Figure 4). MsrA, MsrB or the natural fusion protein containing both MsrA and MsrB domains, MsrBA, could efficiently reduce MetO in MRPs. For example, 4 μg MRP1 required 0.5 μM MsrA or MsrB for 50% reduction (Figure 4A,B) and could be completely reduced by either MsrBA or a combination of MsrA and MsrB (Figure 4B,C). We also utilized the same assay to examine the Trx-dependent Msr activity and found that Msrs could efficiently use Trx to reduce MRPs. For comparison, the reduction of Cys-free MRPs by Msrs was examined by treatment of oxidized MRP5 with Msrs. Interestingly, besides a complete reduction of oxidized MRP5 by Msrs both in DTT- and Trx-dependent assays, this MRP required less NADPH compared with the reduction of MRP1, which contained 15 Cys. The quantitative reduction of MRPs suggested that MsrA and MsrB could target all MetO in these proteins, although probably with different efficiency.

### Antibodies specific for oxidized MRPs

**Figure 5:**
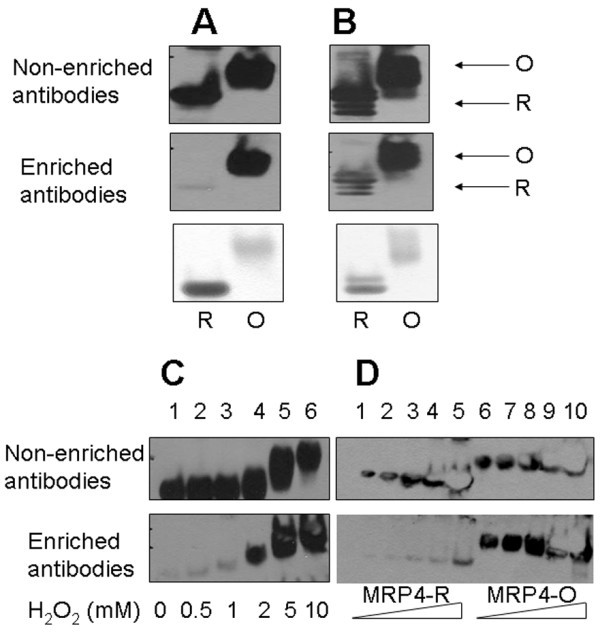
**Immunodetection of MRP in as-isolated and oxidized forms with antibodies enriched for the corresponding oxidized MRPs.** (**A, B**) 2 μg of MRPs (as-isolated and oxidized forms) were resolved on SDS-PAGE gels, transferred onto PVDF membranes, and analyzed by Western blot assays with the antibodies against indicated oxidized MRPs. Top panel: Polyclonal antiserum (non-enriched) used to detect oxidized MRP4 (**A**) or MRP6 (**B**). Middle panel: Polyclonal antiserum against oxidized MRPs was purified on an affinity column containing reduced MRP, and the unbound fraction (designated enriched) was found to be enriched for the antibodies specific for the oxidized MRPs. Bottom panel: protein loading. “R” indicates reduced and “O” indicates oxidized MRPs. (**C**) 2 μg of MRP4 were oxidized with indicated concentrations of H_2_O_2_ for 12 h at room temperature and detected with non-enriched (top panel) or enriched (bottom panel) antibodies for oxidized MRP4 by Western blot analysis. (**D**) Western blot analysis of different amounts of MRP4 oxidized with 10 mM H_2_O_2_ for 12 h at room temperature with non-enriched and enriched antibodies for oxidized MRP4. Triangles represent increasing amounts of MRP loading (0.25, 0.5, 1, 2 and 4 μg). “MRP4-R” indicates the as-isolated MRP4 (lanes 1 to 5), and “MRP4-O” indicates the oxidized MRP4 (lanes 6 to 10).

To generate antibodies recognizing MetO residues, we optimized large-scale MRP oxidation by following mobility of MRPs on SDS-PAGE gels and carrying out mass spectrometry analyses. Antibodies were prepared against all oxidized MRPs, but, as expected, they were found to recognize both reduced and oxidized forms of these proteins. Therefore, we isolated antibodies on affinity columns containing conjugated reduced MRPs, and the flow-through fractions from these columns were found to be enriched for oxidized MRPs. These antibodies were designated e.g. a-MRP4-OX and a-MRP6-OX, for antibodies enriched for oxidized MRP4 and MRP6, respectively (Figure 5A,B). To examine specificity of the enriched antibodies to MRP4, the oxidized MRP4 was immunoblotted with the corresponding antibodies (Figure 5C), which was found to only recognize the protein after it was treated with 2 mM or higher concentrations of H_2_O_2_. Subsequent analyses with reduced and oxidized MRP4 revealed that the antibodies could recognize as little as 20 ng oxidized protein (Figure 5D), whereas the reduced MRP4 could only be detected when the protein amounts were increased to 4 μg.

**Figure 6:**
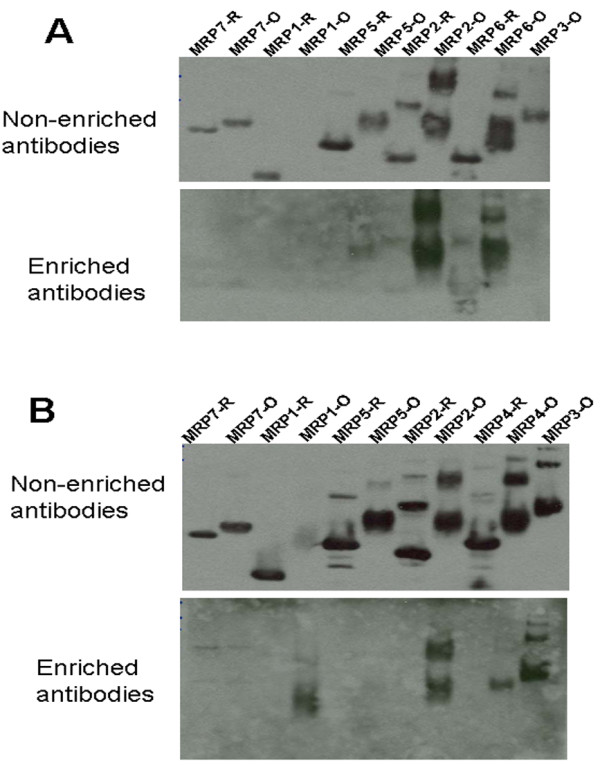
**Antibodies enriched for the oxidized MRPs recognize other oxidized MRPs.** Western blot analysis of as-isolated (MRP-R; R for reduced) and oxidized (MRP-O; O for oxidized) MRPs (MRP1-MRP7) with initial antisera raised against oxidized proteins (top images in each panel) and enriched antibodies (bottom images in each panel). (**A**) Antibodies against oxidized MRP4. (**B**) Antibodies against oxidized MRP6.

**Figure 7:**
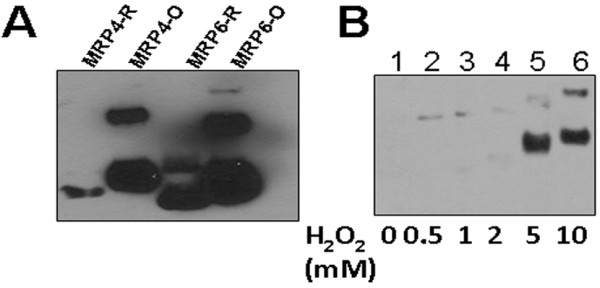
**Enrichment of antibodies for certain oxidized MRPs using affinity columns with other MRPs.** (**A**) Western blot analysis of as-isolated and oxidized (10 mM H_2_O_2_, 12 h) MRP4 and MRP6 with antiserum against oxidized MRP3. “R” indicates as-isolated (i.e., reduced) protein, and “O” indicates oxidized protein. (**B**) Polyclonal antiserum against MRP3 was first purified on a protein G column, then fractionated on an affinity column with as-isolated MRP4, and the unbound fraction was further purified on a column with oxidized MRP4. The bound fraction from the latter column was used for detection of MRP4 oxidized under various H_2_O_2_ treatment conditions.

To test if antibodies enriched for certain oxidized MRPs reacted with other oxidized MRPs, the antibodies enriched for oxidized MRP4 and MRP6 were used to probe various MRPs in both oxidized and reduced forms; the enriched a-MRP4-OX antibodies recognized oxidized MRP2, MRP5 and MRP6, but did not interact with any of the reduced MRPs (Figure 6A). The enriched antibodies to oxidized MRP6 recognized oxidized MRP1, MRP2, MRP3, MRP4, and were unable to detect reduced MRPs. However, non-enriched antibodies interacted with both reduced and oxidized forms of MRPs (Figure 6B). Similarly, non-enriched antibodies to MRP3 detected reduced and oxidized MRP4 and MRP6 (Figure 7A). Based on the observed cross reactivity with MRP4, the antibodies to MRP3 were first applied to the affinity column containing reduced MRP4, and the unbound fraction was further fractionated on the column containing oxidized MRP4; this time the bound and eluted fraction was collected. These antibodies specifically recognized oxidized MRP4 generated by treatment with 5 mM (or higher concentration) H_2_O_2_. However, they did not detect reduced MRP4 or the same protein treated with lower H_2_O_2_ concentrations (Figure 7B).

**Figure 8:**
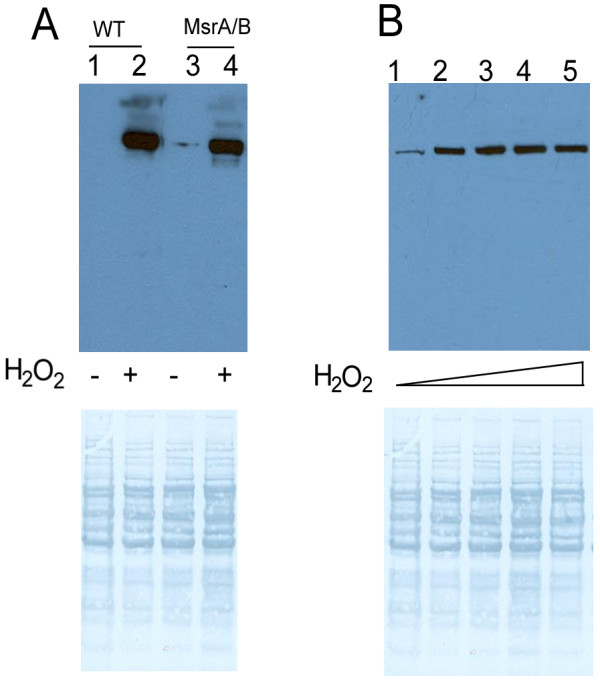
**Antibodies enriched for an oxidized MRP detect a candidate oxidized yeast protein.** Lysates of log-phase yeast cells (BY4741 strain), including wild-type (WT) and MsrA/MsrB double knockout (MsrA/B) strains, were treated with H_2_O_2_, and 30 μg proteins were subjected to Western blot analysis with the antibodies raised against MRP3 that were enriched for the oxidized MRP4 as described in Materials and Methods. (**A**) Yeast lysates were incubated with (lanes 2 and 4) or without (lanes 1 and 3) 100 mM H_2_O_2_ for 1 h at 37°C. (**B**) Wild-type (BY4741) lysates were incubated without (lane 1) or with different concentrations of H_2_O_2_ (lanes 2 to 5) for 1 h at 37°C. The triangles represent increasing amounts of H_2_O_2_ in the oxidation reaction (5, 10, 50, or 100 mM).

Finally, we tested if the resulting antibodies could recognize MetO-containing proteins in yeast extracts. The antibodies made against oxidized MRP3 and further enriched on the MRP4 column (Figure 7B) detected a 62 kDa protein band in the lysate of *S. cerevisiae* BY4741 cells treated with H_2_O_2_ as well as in MsrA/MsrB double knockout lysates treated with H_2_O_2_ (Figure 8). However, this protein band showed similar intensity regardless of occurrence of Msrs and its intensity did not increase with the increase in peroxide concentration. We interpret these data to mean limited utility of MRP antibodies for detection of MetO in endogenous proteins. We also employed other approaches, but were not able to generate antibodies that generally recognize MetO. This finding is consistent with a recent article that reported that the previously reported MetO antibodies do not recognize MetO in proteins [24].

## Discussion

In this work, we characterized Met oxidation and MetO reduction in proteins by SDS-PAGE and immunoblot analyses using novel, second-generation MRPs as substrates. We also developed antibodies specific for oxidized MRPs that could recognize other oxidized MRPs. A key feature of new proteins identified and characterized in the current study is that they contained more than 20% Met, while lacking Cys, whose oxidation may interfere with certain redox assays involving Met oxidation. The use of Cys-free MRPs avoids a complication of variable oxidation of sulfur-containing amino acids [25-29]: in new MRPs proteins, all oxidation events (under relatively mild oxidizing conditions) correspond to Met oxidation.

Whereas previous studies already showed that Met oxidation may result in a decreased mobility of proteins on SDS-PAGE gels, these studies involved a limited number of proteins, and in some cases, no changes in mobility were observed. The use of 7 MRPs, as reported in our study, demonstrates that the decreased mobility on SDS-PAGE gels is a basic property of Met oxidation. Since each of the MRPs has many Met residues, these proteins could be used to monitor Met oxidation and MetO reduction and to assess the degree of Met oxidation at any given point in the assay.

Interestingly, retardation of migration of oxidized MRPs on SDS-PAGE gels was more pronounced than that expected based on the mass difference between reduced and oxidized proteins. For example, a complete oxidation of Met residues in MRP1 increased the protein mass by 0.6 kDa; however, comparison of migration of this protein with protein standards revealed an apparent 6 kDa shift in mobility. Most Met residues in MRPs could be oxidized to MetO by treatment with 10 mM H_2_O_2_ for 12 h at room temperature, and there was an excellent correspondence between mass-spectrometry and SDS-PAGE analyses. Overall, the use of multiple MRPs allows us to state that Met oxidation that results in slower migration of these proteins on SDS-PAGE gels is a general feature of MRPs, and that it is independent of the occurrence of Cys in these proteins.

Although our data suggested that all Met residues could be oxidized by peroxide and targeted for reduction by Msrs, we observed important differences with regard to efficiency of these processes. For example, the use of H_2_O_2_ concentrations above 10 mM typically did not further change MRP migration on SDS-PAGE gels (because all Met were already oxidized at this concentration). However, calmodulin (it contained fewer Met than other MRPs examined in our study) required approximately 50 mM H_2_O_2_ for full oxidation. These data are also consistent with the observations in a previous report [23] and suggest a role of protein structure in protecting Met from oxidation. The sequence context of Met probably also has an effect on Met oxidation in this protein.

The conversion of Met to MetO involves a two-electron oxidation process [30,31]. We further employed MRPs to assess Met oxidation upon treatment with several one- and two-electron oxidants. MRP5 was insensitive to oxidation by CuSO_4_ and FeCl_3_, and the mobility of MRP2 also did not change when it was treated with 25 μM CuCl_2_ and 25 mM ascorbate for 1 h. With regard to two-electron oxidants, H_2_O_2_ being a milder oxidant was more specific than NaOCl in oxidizing Met, whereas a NaOCl oxidized Met at lower concentrations. NaOCl generates HOCl, which is a strong microbicidal agent, capable of killing bacteria, fungi and viruses, and it can modify various residues in proteins, especially Cys, Met, Trp, and Tyr [32,33]. Protein carbonyls can subsequently be generated from chloramines via the loss of HCl and hydrolysis of the amine [34]. We found that at the ratio of NaOCl to MRP 50:1, most Met residues in MRP5 were oxidized. However, when the ratio increased to 250 fold, the amount of MRP5 decreased due to protein degradation. Thus, MRPs are a convenient tool for examining specificity and efficiency of Met oxidation in proteins.

Another application of MRPs is the use of these proteins as substrates in Msr activity assays. We previously found that oxidized MRPs can be reduced by Msrs in a DTT-dependent assay. In the current work, we utilized Trx-dependent reactions involving MsrA, MsrB and a fusion protein MsrBA and found that this assay is equally amenable to the analysis of MetO reduction in MRPs.

Protein state- and modification-specific antibodies, both monoclonal and polyclonal, have been a great instrument in protein analysis, and this especially applies to phosphorylation state-specific antibodies [35]. These reagents opened many exciting opportunities in signaling and regulation studies as well as in diagnostic pathology. Since MetO chemically differs from Met, an attractive possibility is to develop antibodies specific for MetO-containing proteins. However, previous antibodies that recognize MetO have been limited to certain MetO-containing peptides [13,16]. One difficulty in preparing MetO-specific antibodies is that MetO residues in the antigen may be reduced back to Met in the immunized animals. Among antibodies generated against the seven MRPs examined in the current study, we found that the original antisera against MRP3, MRP4 and MRP6 could be enriched for fractions specific for their respective MetO-containing forms by trapping the antibodies that bind the reduced forms of MRPs. We further purified the antibodies on the columns containing other crosslinked oxidized MRPs. The resulting antibodies recognized other MetO-containing proteins.

We tested a potential application of detecting MetO residues in proteins with the antibodies against various MRPs. MRP3 antibodies enriched on the resin with the oxidized MRP4 could recognize a 62 kDa protein band in yeast lysates treated with H_2_O_2_. Since this protein was weakly detected by the same batch of antibodies in the sample not treated with H_2_O_2_, we reasoned that it is sensitive to oxidation. Previously developed antibodies against MRP3 [17] also detected a protein (estimated 55 kDa) in yeast MsrA null cells treated with H_2_O_2_. However, in our hands, this band was not regulated by MsrA/MsrB status and was not increased by treatment with higher concentration of peroxide. More generally, none of the antibodies we developed were able to generally recognize MetO in proteins. This is also consistent with a recent report [24].

## Conclusions

MRPs represent a convenient tool to study Met oxidation and MetO reduction. These proteins and antibodies against them could be used in a variety of applications, several of which are highlighted in our study. MetO can be formed under physiological conditions in cells, but due to low abundance of Met residues and their random oxidation, the analyses of Met oxidation and MetO reduction have been difficult. With the second-generation MRP tools, some of the outstanding questions in Met oxidation and MetO reduction processes can be addressed.

## Abbreviations

Met: Methionine; MRP: Met-rich protein; MetO: Methionine sulfoxide; Msr: Methionine sulfoxide reductase; Cys: Cysteine; DTT: Dithiothreitol; Trx: Thioredoxin.

## Competing interests

The authors declare they have no competing interests.

## Authors' contributions

Designed experiments: XL, YZ, VNG. Carried out research: XL, AK, YZ, DTL, DH. Wrote paper: XL, VNG. All authors read and approved the final manuscript.
